# Draft genome sequence of *Enterobacter hormaechei* HKEH-1 isolated from a diabetic patient’s blood

**DOI:** 10.1128/mra.00452-25

**Published:** 2025-08-11

**Authors:** Mohan Ding, Wuen Ee Foong, Jiabin Huang, Heng-Keat Tam

**Affiliations:** 1 Department of Medical Microbiology, Hunan Provincial Key Laboratory for Special Pathogens Prevention and Control, Hengyang Medical School, University of South China 159373, Hengyang, Hunan, China; 2 Department of Biochemistry and Molecular Biology, Hengyang Medical School, University of South China 159373, Hengyang, Hunan, China; 3 National Health Commission Key Laboratory of Birth Defect Research and Prevention, Hunan Provincial Maternal and Child Health Care Hospital 117925, Changsha, Hunan, China; 4 Institute of Medical Microbiology, Virology and Hygiene, University Medical Center Hamburg-Eppendorf, Hamburg, Germany; University of Maryland School of Medicine, Baltimore, Maryland, USA

**Keywords:** *Enterobacter hormaechei*, plasmids, *Enterobacter cloacae* complex, whole genome sequencing, antibiotic resistance

## Abstract

*Enterobacter hormaechei* is an emerging gram-negative bacterium belonging to the *Enterobacter cloacae* complex. Here, we present the draft genome sequence of the HKEH-1 strain isolated from a blood sample from a type II diabetic patient, harboring three plasmids which share high homology to known plasmids of the *Enterobacter* species.

## ANNOUNCEMENT

*Enterobacter hormaechei* has recently emerged as a common and virulent nosocomial pathogen within the *Enterobacter cloacae* complex (ECC) (1), attributed to its multidrug resistance and the presence of extensive pathogenicity islands (2–4). Although frequently isolated from clinical specimens such as wound exudates, respiratory secretions, and blood (4–6), *E. hormaechei* remains relatively understudied compared to other ECC members. Given its increasing clinical significance and limited characterization, we performed whole-genome sequencing of the *E. hormaechei* HKEH-1 strain to elucidate its genetic features and gain insights into the molecular mechanism underlying multidrug resistance and virulence in hospital-acquired infections.

A bacterial isolate from the hospital’s clinical culture collection was obtained from the blood sample of a hospitalized type II diabetic patient with suspected bacteremia and initially cultured on blood agar at 37°C for 18–24 hours. Based on phenotypic traits, it was presumptively identified as *Acinetobacter baumannii* and sent to the University of South China for molecular analysis. However, sub-culturing on MacConkey agar yielded pink lactose-fermenting colonies, which are uncharacteristic of *A. baumannii* (7, 8). Whole-genome sequencing later confirmed the organism as *Enterobacter hormaechei*, correcting the misidentification.

Genomic DNA was extracted using the standard phenol-chloroform method (9) and fragmented to ~350 bp with a Covaris LE220R-plus system (Covaris, USA). A DNA library was prepared using the Rapid Plus DNA Lib Prep kit (ABclonal Technology, USA) and sequenced with 2×150 bp paired-end reads on an Illumina NovaSeq platform by Novogene (Beijing, China). Sequencing produced 18,254,594 reads. Reads were quality-trimmed using Trimmomatic v.0.39 (10) and assembled *de novo* with SPAdes v.3.15.5 (11), yielding 73 contigs. Genome annotation was carried out using the NCBI Prokaryotic Genome Annotation Pipeline (12). Sequencing and genome assembly statistics are summarized in Table 1. OrthoANIu v.1.2 (13) analysis against the complete genome of *E. hormaechei* strain K528 (GenBank: CP095177) yielded 99.17% identity, exceeding the species cutoff (~95–96%) (14, 15) and supporting species-level classification. Whole-genome alignment with progressiveMauve (build date: 15 May 2023) (16) revealed multiple large locally collinear blocks despite the draft genome’s fragmentation into 73 contigs, confirming strong synteny and conserved genome structure relative to the complete reference. All contigs more than 500 bp were successfully scaffolded to the draft genome using Multi-CSAR v1.1 (17), resulting in a scaffolded assembly of the chromosome totaling 4,506,689 bp. Three contigs remained unscaffolded; mate-pair read mapping at their termini confirmed they are circular plasmids. These plasmids in the HKEH-1 strain show high sequence similarity to known plasmids: 99.52% to p233-2 from *E. hormaechei* strain Y233, 99.26% to pECL-90-4 from *E. hormaechei* strain NJGLYY90-CR, and 99.97% to pECC-247-3 from *E. roggenkampii* strain ECC-247, suggesting possible horizontal gene transfer among *Enterobacter* species. The overall G + C content of the genome was 55.36%. Genome annotation predicted 4,197 coding sequences among a total of 4,374 genes. Antimicrobial resistance gene profiling using ABRicate v.0.3 (https://github.com/tseemann/abricate), with reference to the MEGARes v.2.00 and Comprehensive Antibiotic Resistance Database v3.2.8, identified a total of 32 resistance genes (18, 19). Default parameters were used for all software tools unless otherwise noted.

**TABLE 1:** Summary of sequence data and genome features

Metrics^*a*^	Value
Genome size (bp)	4,519,530
Contig count	73
Total number of reads sequenced	9,127,297 × 2
Coverage depth (sequencing depth)	589.4×
Coarse consistency (%)	99.4
Fine consistency (%)	98.2
Completeness (%)	100
Contamination (%)	0.1
Contigs N50 (bp)	316,426
Contigs L50	4
Guanine-cytosine content (%)	55.36
Number of genes	4,374
Number of coding sequences (CDSs)	4,197
Number of tRNAs	76
Number of rRNAs	4

^*a*^ Genome completeness represents the fraction of universal single-copy functional roles expected for a given taxonomic lineage that are detected in the genome; missing roles indicate incomplete genome assembly or annotation. Contamination is inferred from the detection of multiple copies of these roles, which are expected to be single-copy, suggesting potential contamination or strain heterogeneity. Coarse consistency measures whether the expected functional roles are present or absent as predicted, reflecting broad agreement in the genome annotation. Fine consistency measures whether the number of copies of each functional role matches what is expected based on the genome’s overall pattern, identifying small differences in gene counts. These metrics were calculated using subtools of the BV-BRC platform v3.51.7 (20): EvalG, which estimates genome completeness and contamination based on lineage-specific single-copy marker roles; and EvalCon, which assesses coarse and fine consistency using a machine-learning-derived catalog of approximately 1,300 functional roles with predictable relationships. Coverage depth (sequencing depth) refers to the average number of reads covering each base across the assembled genome, calculated as the total number of clean bases divided by the genome size.

## Data Availability

The whole-genome sequencing project of *E. hormaechei* HKEH-1 has been deposited at NCBI’s GenBank under BioProject accession number PRJNA1255529, and BioSample accession number SAMN48146807. The raw sequences have been deposited in the SRA under the accession number SRX28891146. *E. hormaechei* HKEH-1 whole genome sequence of chromosome and plasmids were deposited to NCBI’s GenBank under the accession numbers CP191206, CP191207, CP191208, and CP191209, respectively.
